# The effect of interventions targeting screen time reduction: A systematic review and meta-analysis

**DOI:** 10.1097/MD.0000000000004029

**Published:** 2016-07-08

**Authors:** Lei Wu, Samio Sun, Yao He, Bin Jiang

**Affiliations:** a Department of Epidemiology, Institute of Geriatrics, Beijing Key Laboratory of Aging and Geriatrics, Chinese People's Liberation Army General Hospital, Beijing, China; b Department of Bioengineering, The University of Tokyo, Tokyo, Japan; c Department of Nanomedicine, Houston Methodist Research Institute, Houston, TX; d State Key Laboratory of Kidney Disease; e Department of Acupuncture, Chinese People's Liberation Army General Hospital, Beijing, China.

**Keywords:** body mass index, meta-analysis, randomized controlled trial, screen time

## Abstract

Previous studies have evaluated the effectiveness of interventions aimed at screen time reduction, but the results have been inconsistent. We therefore conducted a systematic review and meta-analysis of randomized controlled trials (RCTs) to summarize the accumulating evidence of the impact of interventions targeting screen time reduction on body mass index (BMI) reduction and screen time reduction. The PubMed, Embase, and Cochrane Central Register of Controlled Trials (CENTRAL) databases were searched for RCTs on the effect of interventions targeting screen time reduction. The primary and secondary outcomes were the mean difference between the treatment and control groups in the changes in BMI and changes in screen viewing time. A random effects model was used to calculate the pooled mean differences. Fourteen trials including 2238 participants were assessed. The pooled analysis suggested that interventions targeting screen time reduction had a significant effect on BMI reduction (−0.15 kg/m^2^, *P* < 0.001, *I*^2^ = 0) and on screen time reduction (−4.63 h/w, *P* = 0.003, *I*^2^ = 94.6%). Subgroup analysis showed that a significant effect of screen time reduction was observed in studies in which the duration of intervention was <7 months and that the types of interventions in those studies were health promotion curricula or counseling. Interventions for screen time reduction might be effective in reducing screen time and preventing excess weight. Further rigorous investigations with larger samples and longer follow-up periods are still needed to evaluate the efficacy of screen time reduction both in children and in adults.

## Abstract 2

Supplemental Digital Content is available in the text

## 1 Introduction

In recent years, sedentary behavior has emerged as an important risk factor for weight gain, all-cause mortality, and chronic diseases (e.g., cancer, cardiovascular disease, and diabetes).^[1–4]^ The most commonly reported sedentary behavior, apart from occupation, is screen time (time spent in front of a screen) in many populations.^[5–7]^ Screen viewing, including television or video viewing, computer use, and video games, not only increases caloric intake because individuals eat in front of the screen but also negatively influence meal selection through by the power of advertising.^[8]^ In the United States, the prevalence of obesity in participants aged 2 to 19 years increased by 182% in recent decades.^[9]^ Furthermore, obesity and overweight have consistently been regarded as risk factors for a series of negative outcomes.^[10,11]^ In recent years, decreasing screen time has been considered an important public health issue with respect to preventing obesity and overweight and the related disease burden around the world.^[12]^

Previous qualitative reviews^[12–15]^ and meta-analyses^[16–18]^ have summarized interventions for preventing obesity in adults and children. However, the effect of interventions specifically targeting screen time reduction has not been well described. Additionally, the results from studies of the effectiveness of interventions aimed at screen time reduction are inconsistent. Due to the wide variety of methods, outcomes, and measures, 2 qualitative reviews of this topic were performed instead of meta-analyses.^[19,20]^ A meta-analysis by Wahi et al^[21]^ examined the effectiveness of interventions aimed at reducing screen time in children, but the results did not reach statistical significance. In another meta-analysis including both randomized and nonrandomized studies, Maniccia et al^[22]^ concluded that interventions to reduce screen time have a statistically significant effect among children. In recent years, an increasing number of epidemiological studies have been published since the most recent meta-analyses.^[23–27]^ The objective of this study was therefore to summarize the accumulated evidence of the impact of interventions targeting screen time reduction on BMI reduction and screen time reduction in a systematic review and meta-analysis of randomized controlled trials (RCTs) performed with adults and children.

## 2 Methods

### 2.1 Literature search

The present systematic review and meta-analysis was conducted in adherence with the (Preferred Reporting Items for Systematic Reviews and Meta-Analyses—PRISMA) guideline. We searched the PubMed, Embase, and Cochrane Central Register of Controlled Trials (CENTRAL) databases for records to examine the effect of interventions targeting screen time reductions on obesity prevention and screen time reduction with no language restrictions. Our search included the following terms for screen time: “TV,” “television,” “screen time,” “video∗,” and “computer∗.” Details of the search strategy are shown in Appendix 1. The last search was performed on August 24, 2015. The reference lists of relevant studies were searched manually to identify other potentially eligible studies.

### 2.2 Selection criteria

Wu and Sun independently carried out the initial search. Duplicate records were deleted, and the titles and abstracts of each study were screened. We independently identified each study as being excluded or requiring further assessment. Any disagreements were resolved by consensus with the third author (Y. He).

We included studies that met the following criteria: the main aim of the intervention included reducing screen time, and studies containing coinventions could be included; the outcome of the studies referred to changes in screen time and/or changes in BMI, irrespective of whether these were the primary endpoints; and the study design was based on RCTs.

### 2.3 Data extraction

The data extraction was independently performed by Wu and Sun. The following data were extracted from each study: the first author, the date and place of publication, the number of participants enrolled, the recruitment setting, participant characteristics, a summary of the intervention conditions, and the duration of follow-up. The extracted data were entered into a standardized Excel (Microsoft Corporation, Seattle, WA) file. Any disagreements were resolved in discussions with a third author (Y. He).

The primary outcome was the unadjusted mean difference between the treatment and control groups in their changes in body mass index (BMI) from baseline to the longest follow-up time point. The secondary outcome was the mean difference in the changes in screen time (hours per week). All data from each trial with the available mean differences were extrapolated to represent the mean hours per week when possible (e.g., 8.57 m/d = 1 h/w). If the results were not reported in the form of means (95% confidence intervals (CIs)), we used the published literature to identify correlation coefficients to calculate these values. We assumed a correlation coefficient of 0.9 for BMI and 0.5 for television viewing time based on the method reported by Harris et al. and Higgins et al.^[28,29]^ In each trial, the available objectively measured outcomes were used.

### 2.4 Quality assessment

We used the Cochrane Collaboration tool to assess the risk of bias in each study. A “high,” “low,” or “unclear” risk of bias was assigned according to the following domains: random sequence generation, allocation concealment, blinding of the participants and personnel, blinding of the outcome assessment, incomplete outcome data, selective reporting and other biases. Disagreements were resolved in discussions between Wu and Sun.

### 2.5 Statistical analysis

We calculated the mean differences with 95% CIs for the continuous outcome data. A random effects model was used to pool the outcome data regardless of heterogeneity by taking into account between-study and within-study differences. Heterogeneity was defined by the *I*^2^ statistic. Studies with an *I*^2^ statistic >50% were identified as having significant heterogeneity. We further performed a subgroup analysis based on prespecified characteristics (size of the study, baseline age, study location, intervention duration, intervention sessions, intervention type, the presence of cointerventions, and follow-up duration) to investigate potential sources of heterogeneity and of the factors influencing the effect of reduced screen time intervention. Meta-regression analyses were performed to determine whether the effect of the screen time reduction intervention on screen time and BMI was influenced by these factors. Sensitivity analyses were performed to explore the influence of a single study on the overall pooled results by omitting one article at every turn.

The presence of publication bias was evaluated using the Begg and Egger tests. The results were considered statistically significant at *P* values <0.05. Stata, version 12.0 (Stata Corp LP, College Station, TX) and Review Manager Software, version 5.2 (The Nordic Cochrane Centre, Copenhagen, Denmark) were used for the statistical analyses. Given that the present study was a systematic review and meta-analysis of published studies, ethical approval or patient consent was not required.

## 3 Results

### 3.1 Study identification and selection

**Figure 1:**
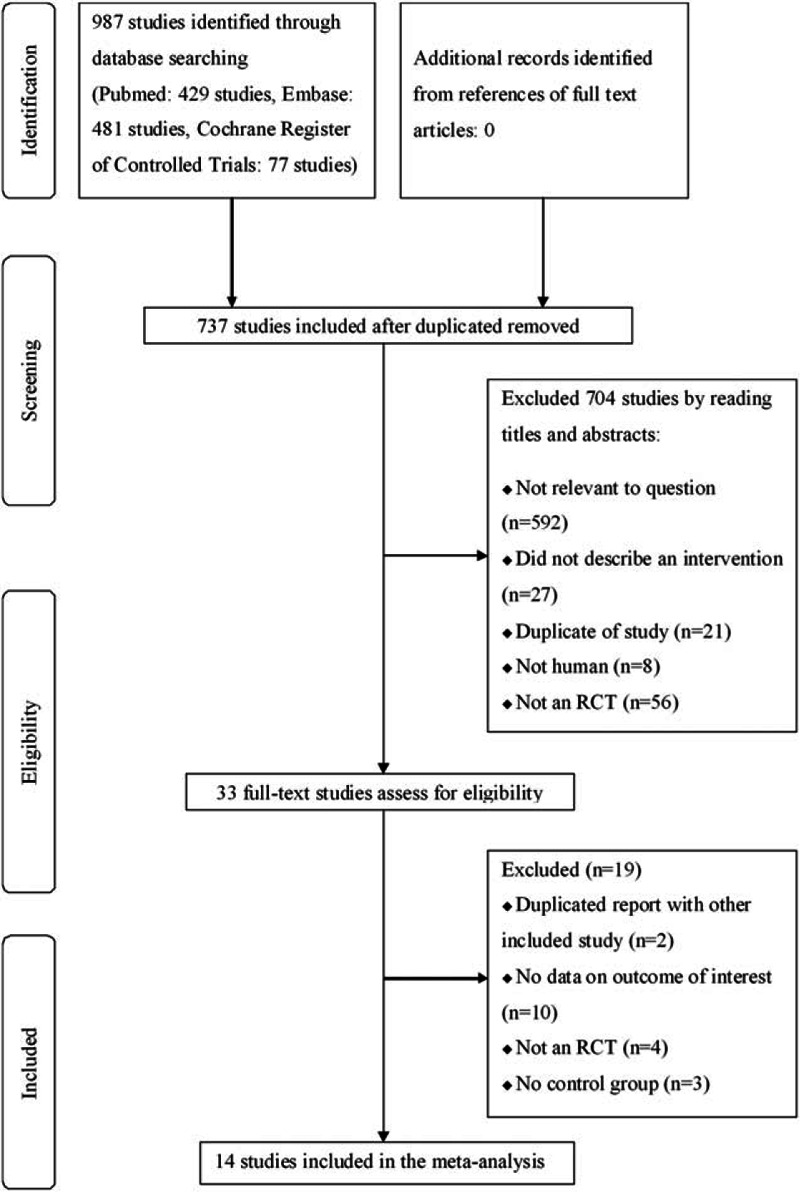
Flow diagram of trials included in the meta-analysis.

A detailed flow diagram of the studies included in the meta-analysis is shown in Fig. 1. A total of 987 studies were identified from the initial database search (PubMed: 429 studies, Embase: 481 studies, Cochrane Register of Controlled Trials: 77 studies). Of these, 250 studies were excluded because they were duplicates, and 704 records were excluded after we read the titles and abstracts. The remaining 33 full-text articles were assessed for eligibility. The reasons for exclusion from the final analysis are as follows: duplicated report with another included study (n = 2), no data on the outcome of interest (n = 10), not an RCT (n = 4), and no control group (n = 3). No additional study was identified through a manual search of the reference lists of the included studies. Finally, 14 studies were included in the present meta-analysis for the secondary outcome.^[23–27,30–38]^ The primary outcome was available in 8 trials.^[24,26,30,34–38]^ We did not include the other 6 trials in the pooled analysis for the primary outcome because 3 trials did not measure BMI,^[25,32,33]^ and 3 trials reported only changes in age- and sex-standardized BMI.^[23,27,31]^

### 3.2 Study characteristics

The main characteristics of the included studies are presented in Table 1. These trials were published between 1999 and 2015. Ten of the included studies were conducted in the United States. The age of the participants ranged from 3 to 54 years. The sample sizes ranged from 21 to 475 (a total of 2238). The duration of the interventions ranged from 3 weeks to 24 months. The follow-up periods ranged from 1 to 24 months. Three of the included studies included other cointerventions (healthy dietary or physical activity interventions).^[26,30,37]^ Seven of the included studies used monitoring devices to assist with allocating screen time or television viewing time.^[24,25,31,33–35,38]^ None of the 14 included trials reported significant differences in the baseline characteristics between the treatment and control groups. In 11 included trials, the control group did not receive any intervention, and the control group in the other 3 trials received intervention via counseling (parents of the child participants),^[23]^ increased physical activity,^[25]^ and verbal advice.^[34]^

**Table 1:**
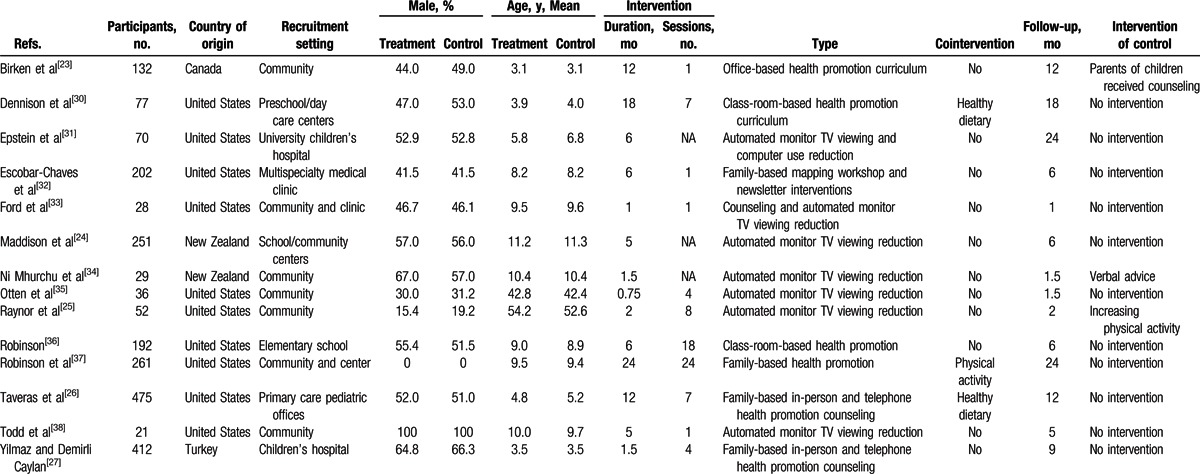
Characteristics of the included trials.

### 3.3 Quality assessment

Appendix 2 summarizes the details of the bias-risk assessment. Twelve trials provided a detailed description of the random sequence generation, and 10 trials reported the appropriate allocation concealment. Six trials reported that the participants and personnel were blinded to the nature of products. Seven trials reported blinding of the outcome assessment. One trial lost more than 20% of the participants in the follow-up period. All of the included trials were judged to have a low risk of reporting bias and other biases.

### 3.4 Primary outcome

**Figure 2:**
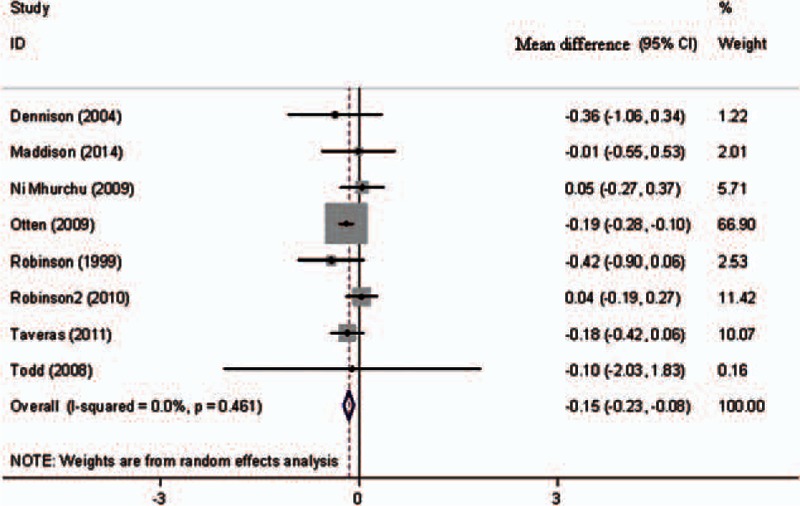
Forest plot of mean differences in body mass index (kg/m^2^).

Eight studies were included in the pooled analysis for the primary outcome (Fig. 2). Compared with the control group, the unadjusted mean BMI difference between the 2 groups was −0.15 kg/m^2^, and the 95% CI was −0.23 to −0.08, with no evidence of significant heterogeneity (*I*^2^ = 0%).

### 3.5 Secondary outcome

**Figure 3:**
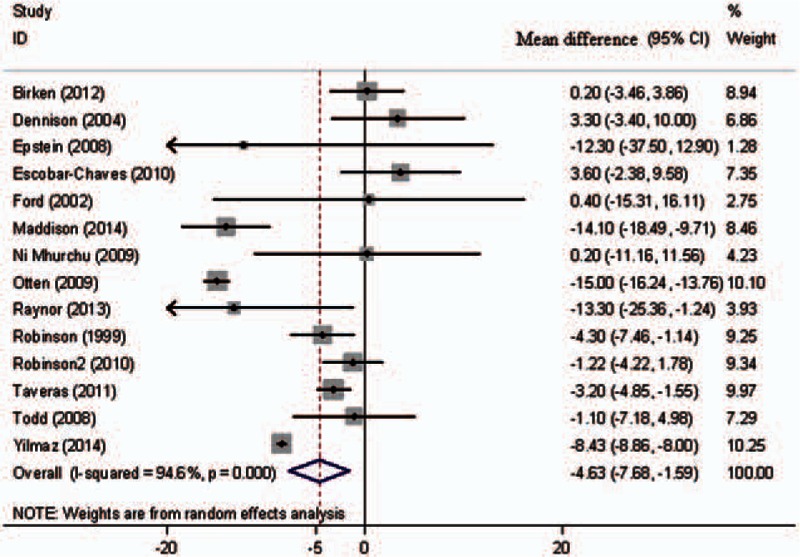
Forest plot of mean differences in screen time (h/w).

Figure 3 presents the pooled analysis of the secondary outcome for 14 studies. Eleven studies reported the amount of screen time. Three other studies reported only the amount of television viewing time but did not include other types of screen viewing time (video viewing, computer use, and video games). We combined the results of the trials for the pooled analysis. The mean difference in screen time (hours per week) between the 2 groups was −4.63, and the 95% CI was −7.68 to −1.59, with evidence of significant heterogeneity (*I*^2^ = 94.6%).

### 3.6 Subgroup analysis

The results of the subgroup analysis of the pooled mean differences in BMI are presented in Table 2. In the subgroup analysis, we did not detect significant differences stratified by the size of the study, baseline age, study location, intervention duration, the number of intervention sessions, intervention type, the presence of cointerventions, and follow-up duration.

**Table 2:**
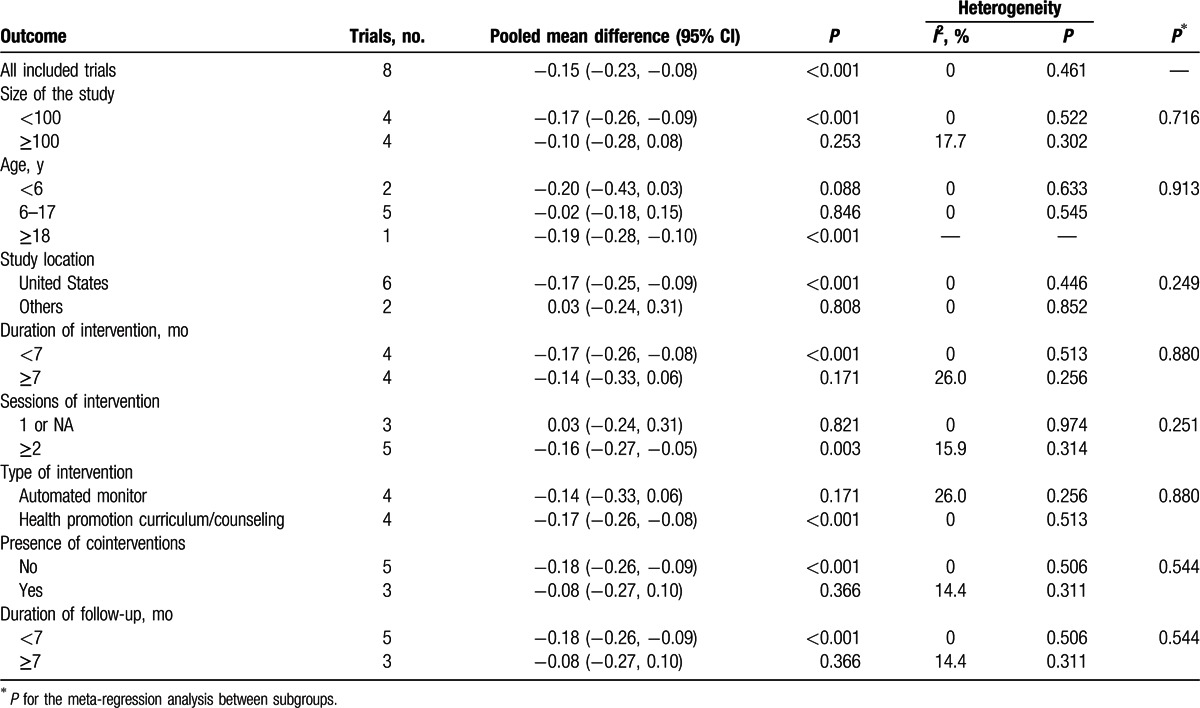
Subgroup analyses of pooled mean differences in body mass index (kg/m^2^).

The results of the subgroup analysis of the pooled mean differences in screen time are presented in Table 3. Significant differences were observed in the subgroup stratified by the duration of intervention (*P* for interaction = 0.010) and type of intervention (*P* for interaction = 0.042). A significant effect of screen time reduction was observed in studies in which the duration of intervention was <7 months (mean difference −8.94, 95% CI −13.17, −4.71) and in studies in which the types of interventions were health promotion curricula or counseling (mean difference −8.76, 95% CI −14.33, −3.19). However, a significant effect was not identified in studies in which the duration of intervention was ≥7 months and in studies that used automated monitoring to assist in reducing screen viewing time.

**Table 3:**
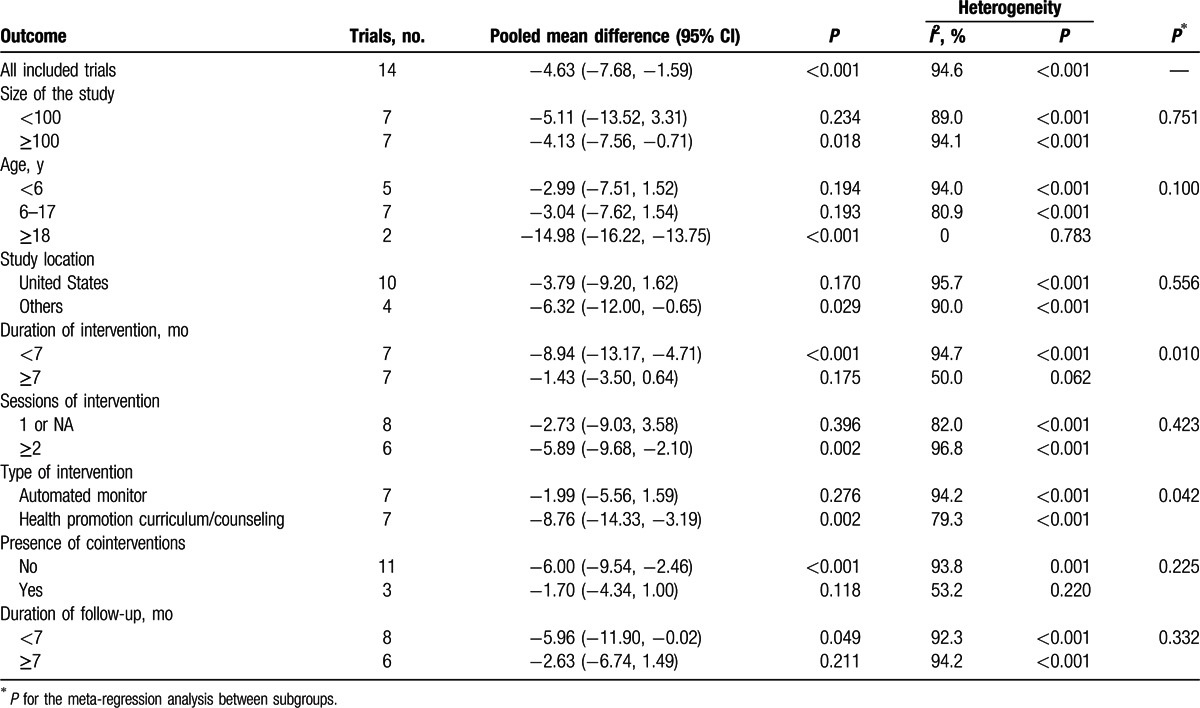
Subgroup analyses of pooled mean differences in screen time (h/w).

### 3.7 Sensitivity analysis

Further exclusion of any single trial significantly altered the overall combined mean difference. The pooled mean difference (95% CI) for BMI ranged from −0.08 (−0.21, 0.06) (excluding a trial by Otten et al^[35]^) to −0.18 (−0.26, −0.09), and the mean difference for screen time ranged from −4.11 (−8.72, 0.49) (excluding a trial by Escobar-Chaves et al^[32]^) to −5.34 (−8.45, −2.23).

### 3.8 Publication bias

For the secondary outcome of screen time, there was no potential publication bias among the 14 included trials (Egger test, *P* = 0.208; Begg test, *P* = 0.228). Publication bias was not assessed for the primary outcome of BMI because the low power with fewer than ten trials limited the interpretability of the findings.^[24]^

## 4 Discussion

The present systematic review and meta-analysis identified 14 trials involving a total of 2238 participants. The pooled analysis of eight trials showed that interventions to reduce screen time had a significant effect on BMI reduction (mean difference −0.15 kg/m^2^, 95% CI −0.23, −0.08), with no evidence of heterogeneity (*I*^2^ = 0%). Furthermore, interventions designed to reduce screen time (14 trials) demonstrated a statistically significant reduction in screen viewing time (mean difference −4.63 h/w, 95% CI −7.68, −1.59), with considerable heterogeneity among the trials (*I*^2^ = 94.6%). A significant effect of screen time reduction was observed in studies in which the duration of intervention was <7 months and in studies in which the types of interventions were health promotion curricula or counseling.

It is biologically plausible that screen time reduction is associated with BMI reduction. Previous studies have reported that screen viewing is often accompanied by the concurrent intake of unhealthy food.^[39,40]^ An unhealthy diet might also be promoted by food advertising on television.^[41,42]^ These factors indicate that screen time reduction might result in changes in dietary behaviors. Additionally, individuals might replace screen viewing time with other physical activities that could contribute to BMI changes.

Differences between our meta-analysis and previous meta-analyses of the same topic should be noted. Consistent with a meta-analysis by Wahi et al,^[21]^ we did not find significant evidence that interventions aimed at reducing screen time effectively reduced BMI and screen time among participants aged 18 years or younger. In another meta-analysis, Maniccia et al^[22]^ showed a small but statistically significant effect on screen time reduction in children. However, a potential bias might have existed due to the inclusion of non-RCTs.^[22]^ Our meta-analysis extends the work of Maniccia et al by reporting the additional outcome of BMI reduction. Additionally, we demonstrated that screen time reduction interventions significantly reduced both BMI and screen time among adults.

Several studies merit individual mention. The results from the investigation by Otten et al^[35]^ showed the largest reduction in BMI. We also observed a statistically significant reduction in screen time in the subgroup analysis of trials that focused on adults.^[25,35]^ These findings suggest that adults may differ from children in how they respond to reductions in screen time. With advancing age, increasing concern about one's health and awareness of the adverse health consequences of screen viewing might motivate adults to establish healthier lifestyles. To the best of our knowledge, limited studies have explored the effects of screen time reduction interventions in adults. The present results revealed that adults were more likely to benefit from screen time reduction interventions. Further investigations should evaluate the efficacy of screen time reduction both in children and in adults.

The intervention method of automated television monitoring devices did not appear to be effective for reducing screen time in the subgroup analysis of the present meta-analysis. In contrast, a qualitative study reported that utilizing an electronic television monitoring device was an effective strategy for reducing television viewing time among young children.^[20]^ A trial using automated television monitoring devices by Ni Mhurchu et al^[34]^ could explain the probable cause of the opposite results. Ni Mhurchu et al found a decrease in self-reported television viewing time in the treatment group that used automated television monitoring devices but found no change in the control group. However, both groups had similar lengths of screen time as assessed by self-reported measurements.^[34]^ These results indicated that participants who used automated television monitoring devices might replace television viewing time with other sedentary screen behaviors. Moreover, a reporting bias might have affected the self- or parent-reported screen viewing time. In the present study, we found that the effect of screen time reduction was significantly only in studies with shorter interventions. One possible explanation is that the effect of the interventions is not maintained over longer interventions.

Our analysis did not include 3 RCTs that tested the effect of multidimensional behavior obesity prevention programs for children.^[42–45]^ The large-scale delivery of multiple health-related interventions might dilute interventions aimed at reducing screen time. Our findings are consistent with those of the above trials that demonstrated small to modest treatment effects for body fat reduction and positive behavior changes. Additionally, we were unable to identify any studies that specifically targeted reductions in media use, including the use of the Internet, mobile phones, and computers. The overall pooled estimates might be significantly affected if trials including these types of media use were included in the pooled analysis.

The current meta-analysis has limitations. First, the major limitation of the present meta-analysis is the significant heterogeneity observed between the included trials. We conducted subgroup analysis and meta-regression to find potential causes of heterogeneity. The significant heterogeneity might be attributed to the type and duration of intervention. In addition, differences in the methods and procedures used to measure BMI and screen time might have also caused the significant heterogeneity in the present meta-analysis. For example, some of the included trials provided electronic monitors to assist in reducing and measuring screen viewing time, whereas others used self- or parent-reported data to measure changes in screen time. The lack of a standardized method to measure screen time might bias the measurement. Future studies would benefit from using a combination of objective and self- or parent-reported screen time measurements. Second, the number of trials included in the present meta-analysis was small, especially studies investigating the primary outcome. Third, half of the included trials had methodological limitations, including a lack of participant blinding and small samples. The possibility of socially desirable responses might affect the real efficacy of interventions. Further high-quality trials with larger samples are needed to confirm our results. Finally, because BMI was the most commonly reported measurement across studies, we used only BMI to measure changes in adiposity. This fact limited our ability to evaluate other important obesity-related measures, such as waist circumference, energy intake, and physical activity.

## 5 Conclusions

In conclusion, the results from the present systematic review and meta-analysis showed that screen time reduction interventions might be effective in reducing screen time and preventing excess weight. Further rigorous investigations with larger samples and longer follow-up periods are still needed to evaluate the efficacy of screen time reduction both in children and in adults. Further studies will be helpful to increase the efficacy of existing strategies and to extend them to different populations.

## Supplementary Material

Supplemental Digital Content
