# Supplementary material for: The effect of interventions targeting screen time reduction: A systematic review and meta-analysis
Source: Medicine (Baltimore). 2016 Jul 8;95(27):e4029. doi: 10.1097/MD.0000000000004029 (PMC5058814; doi:10.1097/MD.0000000000004029)
Supplement: Supplemental Digital Content [file medi-95-e4029-s001.pdf]

APPENDIX 1. Search strategy

Source: PubMed

Searched on: August 27th, 2015

#1 TV [Title/Abstract]

#2 television [Title/Abstract]

#3 “screen time” [Title/Abstract])

#4 video* [Title/Abstract]

#5 computer* [Title/Abstract]

#6 "obesity"[Mesh]

#7 "overweight"[Mesh]

#8 "body mass index"[Mesh]

#9 obes*

#10 overweight*

#11 adiposi*

#12 bmi

#13 random*

#14 "randomized controlled trial"[publication type] OR "randomized controlled trials as topic" [Mesh]

#15 #1 OR #2 OR #3 OR #4 OR #5

#16 #6 OR #7 OR #8 OR #9 OR #10 OR #11 OR #12

#17 #13 OR #14

#18 #15 AND #16 AND #17

Source: Embase

Searched on: August 27th, 2015

#1 TV:ti,ab

#2 television:ti,ab

#3 ‘screen time’:ti,ab

#4 video*:ti,ab

#5 computer*:ti,ab

#6 obesity:ti,ab

#7 obese:ti,ab

#8 ‘body mass index’:ti,ab

#9 bmi:ti,ab

#10 overweight:ti,ab

#11 adiposis:ti,ab

#12 adiposity:ti,ab

#13 random*:ti,ab

#14 ‘randomized controlled trials’:ti,ab

#15 ‘randomized controlled trials’/exp OR ‘randomized controlled trials (topic)’/exp

#16 #1 OR #2 OR #3 OR #4 OR #5

#17 #6 OR #7 OR #8 OR #9 OR #10 OR #11 OR #12

#18 #13 OR #14 OR #15

#19 #16 AND #17 AND #18

Source: Cochrane Central Register of Controlled Trials

Searched on: August 27th, 2015

(TV OR television OR "screen time" OR video OR computer) AND (obesity OR obese OR "body mass index" OR overweight OR adiposis)

	Random sequence generation (selection bias)	Allocation concealment (selection bias)	Blinding of participants and personnel (performance bias)	Blinding of outcome assessment (detection bias)	Incomplete outcome data (attrition bias)	Selective reporting (reporting bias)	Other bias
Birken, 2012	+	+	+	+	+	+	+
Dennison, 2004	+	+	?	+	–	+	+
Epstein, 2008	+	+	?	?	+	+	+
Escobar-Chaves, 2010	?	?	?	?	+	+	+
Ford, 2002	+	+	?	?	+	+	+
Maddison, 2014	+	+	+	+	+	+	+
Ni Mhurchu, 2009	+	?	?	?	+	+	+
Otten, 2009	+	+	+	+	+	+	+
Raynor, 2013	+	+	+	+	+	+	+
Robinson, 1999	?	?	?	+	+	+	+
Robinson, 2010	+	?	?	?	+	+	+
Taveras, 2011	+	+	?	?	+	+	+
Todd, 2008	+	+	+	?	+	+	+
Yilmaz, 2014	+	+	+	+	+	+	+

APPENDIX 2. Assessment of risk of bias of included trials
